# Opening the ICU: views of ICU doctors and nurses before and after liberalization of visiting policies

**DOI:** 10.1186/cc11099

**Published:** 2012-03-20

**Authors:** A Giannini, G Miccinesi, E Prandi, M Audisio, A Bencivinni, E Biagioni, E Castenetto, I Laganà, R Oggioni, V Porta, R Salcuni, A Sarti, MG Visconti, C Borreani

**Affiliations:** 1 Fondazione IRCCS Ca' Granda, Ospedale Maggiore Policlinico, Milan, Italy; 2 Istituto per lo Studio e la Prevenzione Oncologica, Florence, Italy; 3 Ospedale di Ciriè, Italy; 4 Ospedale Nuovo del Mugello, Borgo San Lorenzo, Italy; 5 Azienda Ospedaliera Universitaria di Modena, Italy; 6 Ospedale Civico, Chivasso, Italy; 7 Azienda Ospedaliera 'G. Salesi', Ancona, Italy; 8 Ospedale Civile, Legnano, Italy; 9 Ospedale di Ivrea, Italy; 10 Ospedale S. Maria Nuova, Florence, Italy; 11 Ospedale 'A. Uboldo', Cernusco sul Naviglio, Italy; 12 Istituto Nazionale per lo Studio e la Cura dei Tumori, Milan, Italy

## Introduction

Italian ICUs still impose restrictive visiting policies (with a median visiting time of about 2 hours/day); however, a revision of current policies is underway [1-3]. No data are available on the views of Italian ICU teams following an at least partial liberalization of visiting policies. We investigated this issue in the course of a survey about the impact on ICU teams of the liberalization of visiting policies.

## Methods

We administered an anonymous closed-question questionnaire to nurses and doctors at eight ICUs that were about to increase daily visiting time to at least 8 hours, soliciting their views on policy changes in their unit. The ICU staff were asked to fill in the same questionnaire a year after implementation.

## Results

The first response rate was 91% (234/258), the second 76% (197/258). In the first instance, 83% of doctors and 67% of nurses expressed a favourable opinion regarding the change in visiting policy. After 1 year a positive opinion was expressed by 84% of doctors and 63% of nurses. Both phases of the study show a significant predominance of positive opinions among doctors (*P = *0.032 and 0.005).

## Conclusion

Most ICU staff members view the opening of the unit positively, and on the whole maintain this opinion 1 year after the policy change. Overall, the attitude of doctors is more favourable than that of nurses. It is essential to build up a picture of the difficulties that liberalizing visiting could create for ICU staff (and particularly for nurses), and to explore the causes and extent of such difficulties, in order to identify possible solutions and offer nurses and doctors appropriate support.
